# Functional analysis of OCTN2 and ATB^0,+^ in normal human airway epithelial cells

**DOI:** 10.1371/journal.pone.0228568

**Published:** 2020-02-06

**Authors:** Bianca Maria Rotoli, Rossana Visigalli, Amelia Barilli, Francesca Ferrari, Massimiliano G. Bianchi, Maria Di Lascia, Benedetta Riccardi, Paola Puccini, Valeria Dall’Asta

**Affiliations:** 1 Laboratory of General Pathology, Department of Medicine and Surgery, University of Parma, Via Volturno, Parma, Italy; 2 Preclinical Pharmacokinetics, Biochemistry & Metabolism Department, Chiesi Farmaceutici, Largo F. Belloli, Parma, Italy; Hungarian Academy of Sciences, HUNGARY

## Abstract

In human, OCTN2 (SLC22A5) and ATB^0,+^ (SLC6A14) transporters mediate the uptake of L-carnitine, essential for the transport of fatty acids into mitochondria and the subsequent degradation by β-oxidation. Aim of the present study was to characterize L-carnitine transport in EpiAirway^™^, a 3D organotypic *in vitro* model of primary human tracheal-bronchial epithelial cells that form a fully differentiated, pseudostratified columnar epithelium at air-liquid interface (ALI) condition. In parallel, Calu-3 monolayers grown at ALI for different times (8d or 21d of culture) were used as comparison. OCTN2 transporter was equally expressed in both models and functional at the basolateral side. ATB^0,+^ was, instead, highly expressed and active on the apical membrane of EpiAirway^™^ and only in early-cultures of Calu-3 (8d but not 21d ALI). In both cell models, L-carnitine uptake on the apical side was significantly inhibited by the bronchodilators glycopyrrolate and tiotropium, that hence can be considered substrates of ATB^0,+^; ipratropium was instead effective on the basolateral side, indicating its interaction with OCTN2. Inflammatory stimuli, such as LPS or TNFα, caused an induction of SLC6A14/ATB^0,+^ expression in Calu-3 cells, along with a 2-fold increase of L-carnitine uptake only at the apical side; on the contrary SLC22A5/OCTN2 was not affected. As both OCTN2 and ATB^0,+^, beyond transporting L-carnitine, have a significant potential as delivery systems for drugs, the identification of these transporters in EpiAirway^™^ can open new fields of investigation in the study of drug inhalation and pulmonary delivery.

## Introduction

L-Carnitine (β-hydroxy-γ-trimethylaminobutyrate) is a small, highly polar zwitterionic molecule, essential in the transfer of activated long-chain fatty acids across the inner mitochondrial membrane (the so-called “carnitine shuttle”), for their degradation through β-oxidation [1]. Besides its key role in energy metabolism, several studies provide evidence that L-carnitine also functions as a cytoprotector by promoting cell resistance and antiapoptotic pathways, as well as by enhancing antioxidative resources [2,3,4].

Carnitine availability in humans is modulated by OCTN2 transporter, as clearly suggested by the autosomal recessive disorder “Systemic primary carnitine deficiency” (SPCD; OMIM 212140). Here, mutations of SLC22A5 gene impair L-carnitine absorption in the intestinal tract and increase its loss in the urines, due to the defective renal reabsorption from the glomerular filtrate; the resulting reduction of carnitine circulating levels causes a decrease of intracellular accumulation that impairs fatty acid oxidation, strongly compromising the function of several tissues [1]. Biochemically, OCTN2 operates a Na^+^-dependent, high-affinity (K_m_ is in the range of 10–20 μM) uptake of L-carnitine, the physiological substrate, and other carnitine derivatives, as well as a Na^+^-independent transport of organic cations [5,6,7]. OCTN2 expression has been demonstrated in liver, heart, testis, skeletal muscle, lung and brain, sustaining a role for the transporter in the systemic distribution of carnitine [8]; the transporter has been also found in human macrophages, where it has been identified as a novel target gene of the mTOR‐STAT3 axis [9]. In polarized epithelia, such as intestine and kidney, OCTN2 is located in the apical membrane of the cells [10], where it is involved in intestinal absorption and renal reabsorption. As far as human lung is concerned, OCTN2 expression has been detected in several respiratory epithelial models, such as A549, 16HBE14o^-^, BEAS-2B, NCI-H292, NCI-H441, as well as Calu-3 cells [11,12,13,14,15]. Recently, OCTN2 has been included in the catalog of transporters responsible for the interaction with drugs by the International Transporter Consortium (ITC) [16]; its involvement in the transport of bronchodilators has been also suggested [17,18].

The other transporter involved in carnitine absorption is ATB^0,+^, a system responsible for the Na^+^/Cl^—^dependent influx of neutral and cationic amino acids. This transporter has a low affinity (Km = 800 μM) for L-carnitine, but an high concentrative capacity, being energized by the transmembrane gradients of Na^+^ and Cl^−^, as well as by membrane potential [19]. Consistent with functional studies, ATB^0,+^ is expressed in the lung and intestine under normal conditions [20,21], where it is supposed to be mainly involved in nutrient uptake, due to its broad specificity and concentrative transport mechanisms [22,23].

In a previous contribution we addressed L-carnitine transport in undifferentiated human airway epithelial cells and we demonstrated that OCTN2 is the only transporter active in A549 and BEAS-2B cells, while both OCTN2 and ATB^0,+^ are operative in Calu-3 and NCl-H441 [12]. Although these models are usually employed as reference in studies of pulmonary drug absorption [24], they all are immortalized or transformed cell lines, and criticisms exist as their biological functions can differ from those of primary differentiated human airway epithelial cells. In recent years, three-dimensional systems of human primary airway cells have been developed, reproducing respiratory epithelium *in vivo*. Among these, the EpiAirway^™^ system consists of a pseudostratified epithelium containing the differentiated cell types found in the respiratory epithelium, like mucus-producing goblet cells, ciliated and basal cells; it appears particularly promising and innovative since, grown under air-liquid interface (ALI) conditions, closely resembles the epithelium *in vivo* [25,26]. To date, however, little is known about the biophysical features of this model and, more precisely, only minimal information is available on the membrane transporters responsible for fluxes of nutrients, such as amino acids, sugar, vitamins, and other substances like drugs across the plasma membrane. The purpose of the present study is, hence, to characterize the expression and activity of L-carnitine transporters in EpiAirway^™^, by employing Calu-3 cells grown under ALI conditions as comparison.

## Methods

### Cell cultures

EpiAirway^™^ tissues (AIR-200-PE6.5), supplied by MatTek Corporation (Ashland, MA), were used. Cultured on microporous membrane inserts at the air-liquid interface (ALI), EpiAirway^™^ recapitulates aspects of the *in vivo* microenvironment of the lung; this system is, indeed, produced from primary human tracheal-bronchial epithelial cells that form a fully differentiated, pseudostratified columnar epithelium containing mucus-producing goblet cells, ciliated cells and basal cells. Upon arrival, tissue inserts were transferred to 24-well plates containing 600 μl of the AIR 200-M125 medium and equilibrated overnight at 37°C and 5% CO_2_; medium at the basolateral side was, then, renewed every day, while apical washes for mucus removal were performed employing the solution provided by the manufacturer. Cultures from five different healthy donors were employed.

Calu-3 cells (American Type Culture Collection), obtained from a human lung adenocarcinoma and derived from serous cells of proximal bronchial airways, were cultured in Eagle's Minimum Essential Medium (EMEM) supplemented 10% fetal serum (FBS), sodium pyruvate (1 mM) and 1% penicillin/streptomycin. Cells between passages 25–30 were routinely cultured under physiological conditions (37.5°C, 5% CO_2_, 95% humidity) in 10-cm diameter dishes. For the experiments, Calu-3 cell monolayers were grown at ALI; to this end, 10^5^ cells were seeded onto each Transwell polyester insert (0.33 cm^2^, 0.4 μm pore size; Falcon) and the apical medium was removed 24 hours after seeding, while basolateral medium was renewed every other day. The monolayers were allowed to differentiate under ALI condition over 8 or 21 days.

Where indicated, lipopolysaccharides from E. Coli 0111:B4 (LPS), Tumor Necrosis Factor α (TNFα) or Interleukin 4 (IL-4) were added in complete growth medium for 24 h.

### Trans-epithelial electrical resistance and paracellular permeability

Before each experiment, the integrity of the monolayers was assessed by measuring the trans-epithelial electrical resistance (TEER) using an epithelial voltohmmeter (EVOM, World Precision Instruments). In parallel, the paracellular permeability to extracellular solutes was assessed by measuring the apical-to-basolateral flux of ^14^C-Mannitol (1 μCi/ml corresponding to 20 μM); the apparent permeability coefficient (*P*_*app*_) of mannitol was calculated according to the equation:

$$P_{app}=\left(dQ/dt\right)/\left(A\times C_{0}\times 60\right)$$

where dQ/dt is the transport rate, A is filter surface area (0.33 cm^2^), C_0_ is the initial mannitol concentration, and 60 is the conversion from minutes to seconds. TEER and mannitol *P*_*app*_ values are shown in S1 Fig.

### Carnitine uptake

L-Carnitine transport was measured both at the apical and the basolateral side of cell monolayers. After two washes in pre-warmed transport buffer (Earle's Balanced Salt Solution (EBSS) containing (in mM) 117 NaCl, 1.8 CaCl_2_, 5.3 KCl, 0.9 NaH_2_PO_4_, 0.8 MgSO_4_, 5.5 glucose, 26 Tris/HCl, adjusted to pH 7.4), cells were incubated in fresh transport buffer (100 μl in the apical and 600 μl in the basolateral compartment) containing L-[^3^H]carnitine (2 μCi/ml). Where indicated, the inhibitors or the drugs were added to the transport buffer at the indicated concentrations. In particular, L-carnitine uptake through OCTN2 has been determined employing betaine as specific inhibitor [27], while arginine has been used as selective inhibitor of ATB^0,+^, that is known to accept this amino acid as substrate [22,28,29]. Notably, arginine does not interact with OCTN2 [12] and betaine does not interact with ATB^0,+^ [19]. For a sodium-free EBSS, 117 mM NaCl was replaced with equimolar N-methyl-D-glucamine chloride. After 30 min, transport buffer was removed, and the experiment terminated by two rapid washes (< 10 s) in ice-cold urea (300 mM). The filter was, then, detached from the insert and the ethanol soluble pool was extracted from monolayers; radioactivity in cell extracts was determined with MicroBeta^2^ liquid scintillation spectrometer (Perkin Elmer, Italy). Protein content in monolayers was determined directly in the filters using a modified Lowry procedure [30]. L-carnitine uptake is expressed as pmol/mg of protein/min. In order to discriminate the contribution of OCTN2 and ATB^0,+^ transporters, L-carnitine uptake was measured either in the absence or in the presence of betaine or arginine employed as inhibitors of OCTN2 [12] and ATB^0,+^ [22,28], respectively. Hence, the activity of ATB^0,+^ is calculated from the difference between the uptake measured in the absence and in the presence of arginine, while the activity of OCTN2 results from the difference between the uptake measured in the absence and in the presence of betaine.

### RT-qPCR analysis

Total RNA was isolated with GeneJET RNA Purification Kit and quantified through measurement of the A_260_/A_280_ ratio with NanoDrop2000 Spectrophotometer (Thermo Fisher Scientific); 1μg of RNA was, then, reverse transcribed with RevertAid First Strand cDNA Synthesis Kit (Thermo Fisher Scientific). qPCR was, then, performed on 20ng of cDNA by employing StepOnePlus Real-Time PCR System (Thermo Fisher Scientific). The expression of SLC6A14/ATB^0,+^ (NM_007231.5) was measured using specific forward/reverse primers (5'GCTGCTTGGTTTTGTTTCTCCTTGGTC3' and 5’GCAATTAAAATGCCCCATCCAGCAC3') and SYBR^™^ Green PCR Master Mix (Thermo Fisher Scientific); the amount of SLC22A5/OCTN2 (NM_001308122) and that of the reference gene RPL15 (Ribosomal Protein Like 15, NM_001253379.2) were, instead, monitored employing specific TaqMan® Gene Expression Assays (Thermo Fisher Scientific; Cat# Hs00929869_m1, Lot#1514580, and Hs03855120_g1, Lot# P180525-000A12, respectively), according to the manufacturer’s instructions. The expression of the reference gene was unaffected by any of the experimental treatments adopted. The amount of the genes of interest is calculated relatively to that of the reference gene RPL15 using the formula 1000×2^Δ*Ct*^ (where Δ*Ct* = *Ct*_*RPL*15_−*Ct_gene of interest_*).

### Immunofluorescence staining and analysis with confocal laser scanning microscopy (CLSM)

Calu-3 monolayers and EpiAirway^™^ tissues were rinsed in phosphate buffered saline (PBS), then fixed with ice cold methanol for 7 min or with 3.7% paraformaldehyde at room temperature for 15 min, respectively. Cells were, then, permeabilized with a 20 min incubation in 0.2% Triton X-100 in PBS. After 1h in blocking solution (5% of bovine serum albumin in PBS) at 37°C, cells were incubated overnight at 4°C with the polyclonal antibody ATB^0,+^ or OCTN2 (1:100, Sigma-Aldrich), then washed with PBS and further incubated for 45 min with Alexa Fluor 488 antibody (1:400, Abcam). After three rinses, cells were incubated with propidium iodide solution (Cell Signaling, EuroClone) for 10 min at 37°C, so as to stain nuclei; control filters were stained with propidium iodide in the absence of primary antibodies, so as to obtain only nuclei signal. After staining procedures, the filters were detached from the culture inserts and mounted on microscope slide glass slides with fluorescence mounting medium (FluorSave Reagent, Calbiochem). Samples were observed with a Zeiss® 510 LSM Meta confocal microscope through a 63x (NA 1.4) oil objective, adopting a multi-track detection mode. Excitation at 488 nm and emission recorded through a 505–530 nm band pass barrier were used for the detection of the transporters; excitation at 543 nm and emission recorded through a 580–630 nm band pass barrier filter were adopted for cell nuclei. The two signals were rendered with scales of green and red for transporters and nuclei, respectively. Vertical sections were obtained with the function Display-Cut (Expert Mode) of the LSM 510 confocal microscope software (Microscopy Systems). Reconstructions were performed from z-stacks of digital images, processed with the Axiovision module inside 4D release 4.5 (Carl Zeiss), applying the shadow or the transparency algorithm.

### Statistical analysis

The statistical analysis was performed using GraphPad Prism 7 (GraphPad Software, San Diego, CA, US). Differences in carnitine uptake were analyzed with a two tailed Student's t-test for unpaired data, as well as the effect of inflammatory stimuli on gene expression. The expression of SLC22A5 and SLC6A14 in the different cell models was compared with One-Way ANOVA with Bonferroni Post test. p < 0.05 was considered significant.

### Materials

Fetal bovine serum was purchased from EuroClone (Milano, Italy). Carnitine-L-[N-methyl-^3^H]HCl (80 Ci/mmol) and Mannitol, D-[1-^14^C] (57.2 mCi/mmol) were obtained from Perkin-Elmer (Milano, Italy). Sigma-Aldrich (Milano, Italy) was the source of the antibodies and, unless otherwise specified, of all other chemicals.

## Results

**Fig 1:**
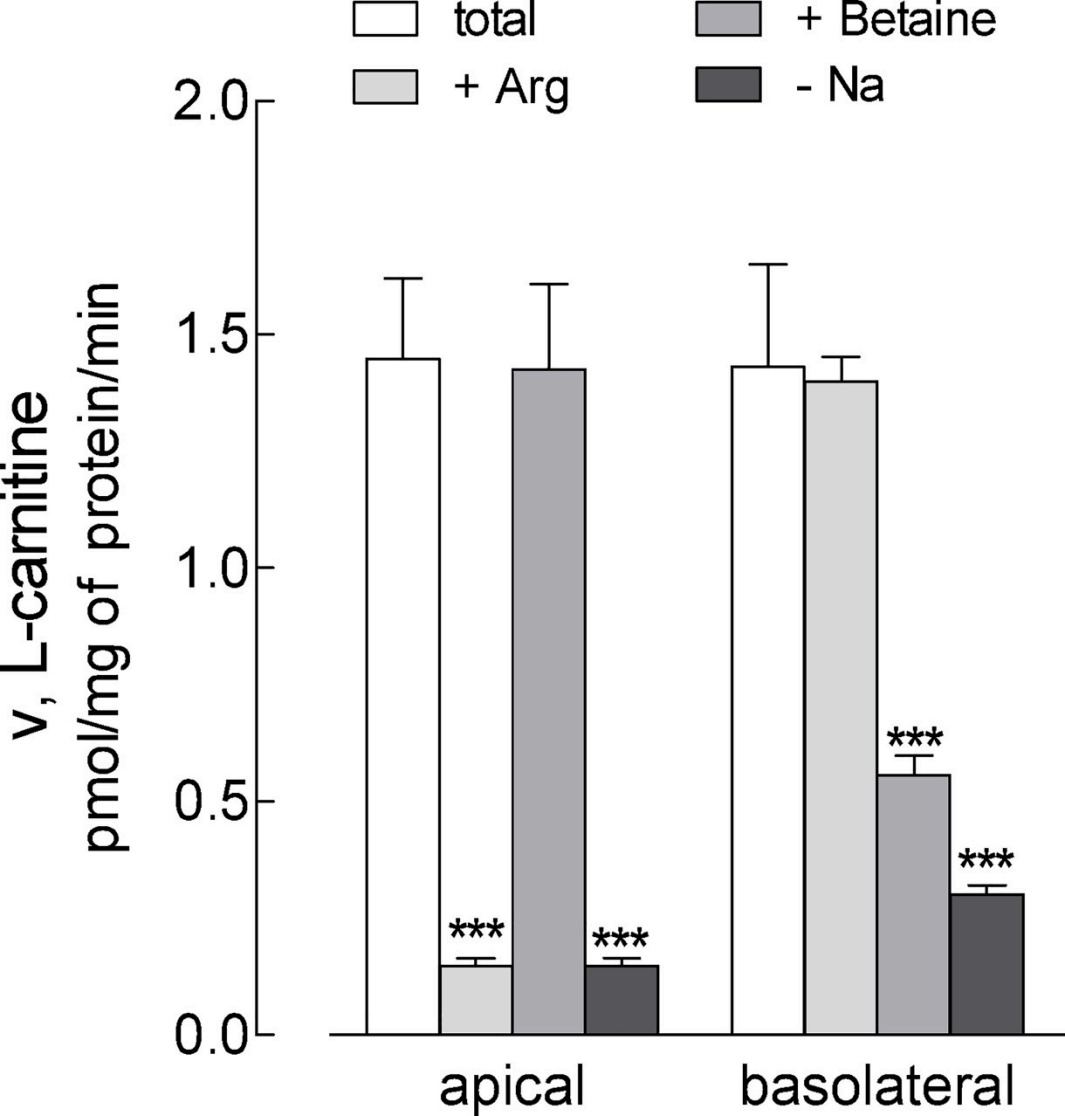
Characterization of carnitine uptake in EpiAirway^™^. Monolayers of EpiAirway^™^ cells grown under air-liquid interface (ALI) conditions were washed with EBSS and incubated for 30 min, either at the apical or the basolateral compartment, in EBSS containing [^3^H]carnitine (1 μM; 2 μCi/ml) in the absence (none) or in the presence of 2 mM arginine or 2 mM betaine, as indicated. For sodium-independent uptake (- Na), a Na^+^-free EBSS was employed (see Methods). Bars represent the mean ± SEM of four independent experiments. **p<0.01, ***p<0.001 vs none. Original data are available at osf.io (DOI 10.17605/OSF.IO/Z3UB5).

In order to address the transporters responsible for L-carnitine transport in EpiAirway^™^, the uptake was measured at both the apical and the basolateral side of the monolayers, either in the absence or in the presence of sodium, by employing inhibitors specific for each transporter (Fig 1). L-Carnitine influx was totally sodium-dependent at both culture sides, excluding the contribution of sodium-independent transport systems. On the apical compartment, the uptake was almost fully (90%) inhibited by arginine, which is known to compete with carnitine for ATB^0,+^ transporter [22,28], while betaine, substrate of OCTN2 [12], was completely ineffective. On the contrary, betaine markedly inhibited L-carnitine transport at the basolateral side, where arginine was, instead, completely ineffective. In light of these findings we can conclude that L-carnitine transport in EpiAirway^™^ is due to the activity of ATB^0,+^ transporter on the apical and OCTN2 at the basolateral side.

**Fig 2:**
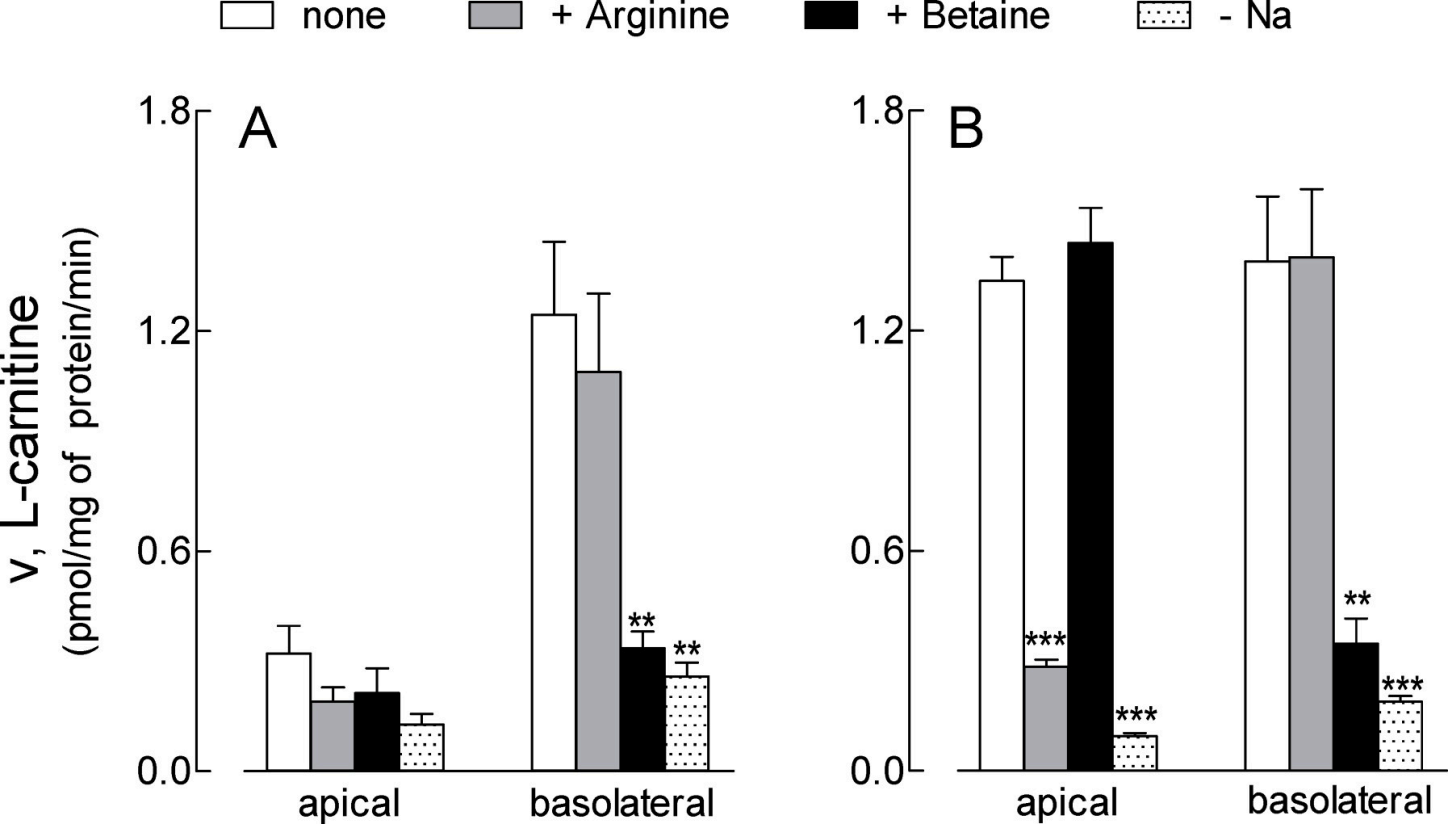
Characterization of carnitine uptake in Calu-3 cells. Monolayers of Calu-3 cells grown under air-liquid interface (ALI) conditions for 21d (A) or 8d (B) were washed with EBSS and L-carnitine uptake was measured as described in Fig 1. Bars represent the mean ± SEM of four independent experiments. **p<0.01, ***p<0.001 vs none. Original data are available at osf.io (DOI 10.17605/OSF.IO/Z3UB5).

The same functional analysis was, then, applied to Calu-3 cells cultured under ALI conditions for 21d (Fig 2, panel A). Unexpectedly, the uptake at the apical side was very low, not significantly inhibited by the presence of either arginine or betaine, and not affected by the absence of sodium. At the basolateral side, on the contrary, transport data were comparable to those obtained in EpiAirway^™^, with L-carnitine influx being totally sodium-dependent and completely inhibited by betaine, but not by arginine. In order to better address the differences observed between the two models, the same analysis was repeated in Calu-3 cells cultured under ALI conditions for a shorter time (8d). In these cells, L- carnitine transport measured at the apical side was higher than in Calu-3 cultured for 21d and comparable to that of EpiAirway^™^, with the same inhibition pattern; similarly, also data obtained at the basolateral side overlapped with those of primary normal cells (Fig 2, panel B). In conclusion, the results of transport analysis demonstrated that L-carnitine uptake at the basolateral side of both EpiAirway^™^ and Calu-3 cells is mediated by OCTN2, while on the apical membrane it depends on the activity of ATB^0,+^; this latter, however, is readily detectable only when Calu-3 are cultured for short times, and decreases along with the differentiation process.

**Fig 3:**
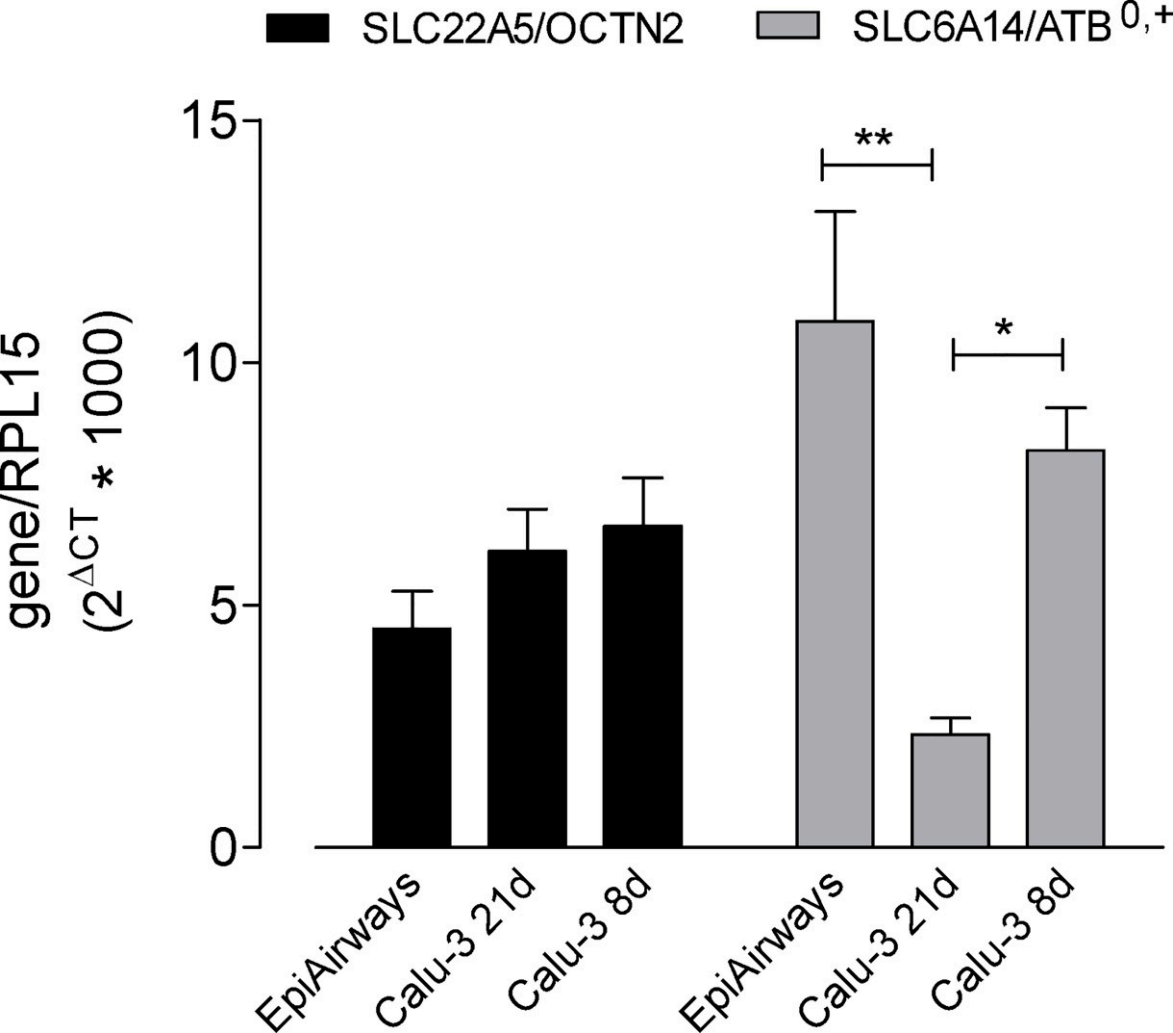
Expression of SLC22A5/OCTN2 and SLC6A14/ATB^0,+^ in EpiArway^™^ and Calu-3 cells. mRNA levels were determined in EpiAirway^™^ and in Calu-3 cultured under ALI conditions for 8d or 21d, as indicated, by means of RT-qPCR analysis. The expression of the gene of interest was shown after normalization for that of the reference gene (RPL15). Data are means ± SEM of five independent experiments. *p<0.05, **p<0.01. Original data are available at osf.io (DOI 10.17605/OSF.IO/Z3UB5).

The expression of the different transporters was consistent with this finding, both at gene and protein level. The mRNA for SLC22A5/OCTN2 was, indeed, equally expressed in EpiAirway^™^ and in Calu-3, both cultured for 8 and 21d; SLC6A14/ATB^0,+^ on the contrary was maximally detectable in EpiAirway^™^, expressed to a lesser extent in Calu-3 at 8d ALI and much less abundant after 21d of culture (Fig 3).

**Fig 4:**
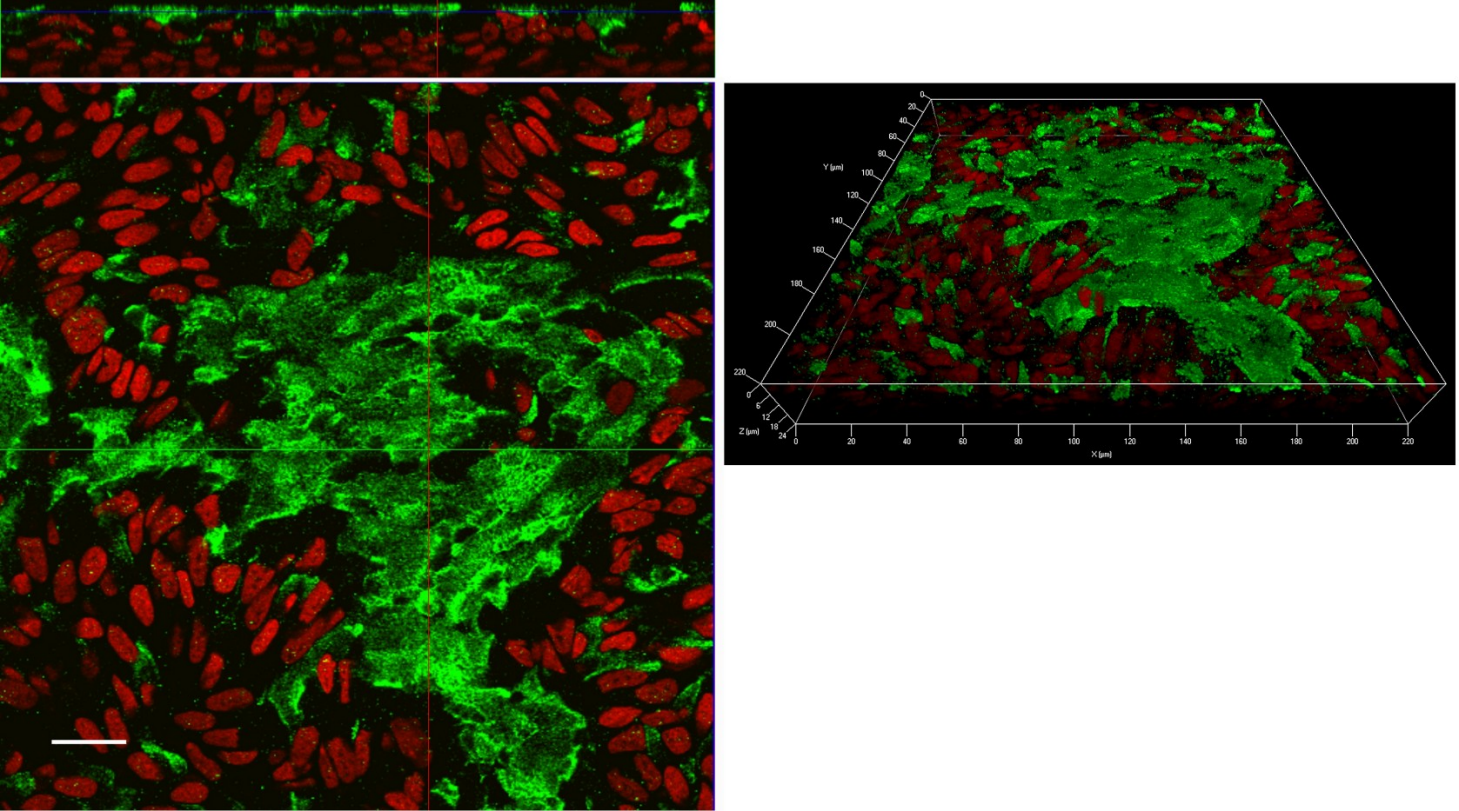
Immunolocalization of ATB^0,+^ transporter in EpiAirway^™^. Confocal laser scanning microscopy of EpiAirway^™^ layers immunolabelled for ATB^0,+^ transporter (green) is shown. Nuclei were stained with propidium iodide (red). Left: single XY scan acquired in correspondence of the apical membrane. Top: XZ section of the plane. Right: 3D reconstruction of z-stacks confocal images; about 30 horizontal sections were acquired. Representative images from three independent experiments are shown. Bar, 20 μm.

**Fig 5:**
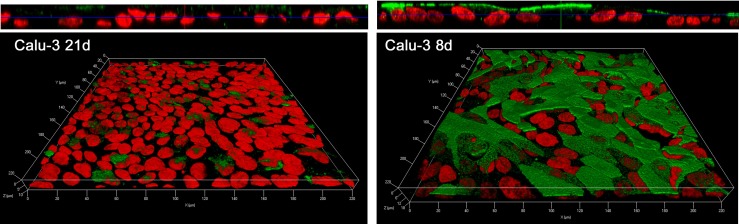
Immunolocalization of ATB^0,+^ transporter in Calu-3 cells. Confocal laser scanning microscopy of Calu-3 monolayers immunolabelled for ATB^0,+^ transporter (green) is shown. Nuclei were stained with propidium iodide (red). Cells were cultured under ALI condition for 21d (left) or 8d (right). Top: XZ scans are shown. Bottom: 3D reconstruction of z-stacks confocal images; about 25 horizontal sections were acquired. Representative images from three independent experiments are shown. Bar, 10 μm.

As for the corresponding proteins, these where analyzed by means of immunocytochemistry. As shown in confocal images, ATB^0,+^ transporter was markedly expressed at the apical side of both EpiAirway^™^ (Fig 4) and Calu-3 monolayers cultured for 8d (Fig 5) with a more homogeneous staining in Calu-3 cells than in EpiAirway^™^, likely due to the pseudostratification of normal airway cell system. The protein was, instead, only barely detectable in Calu-3 after 21d of culture (Fig 5).

**Fig 6:**
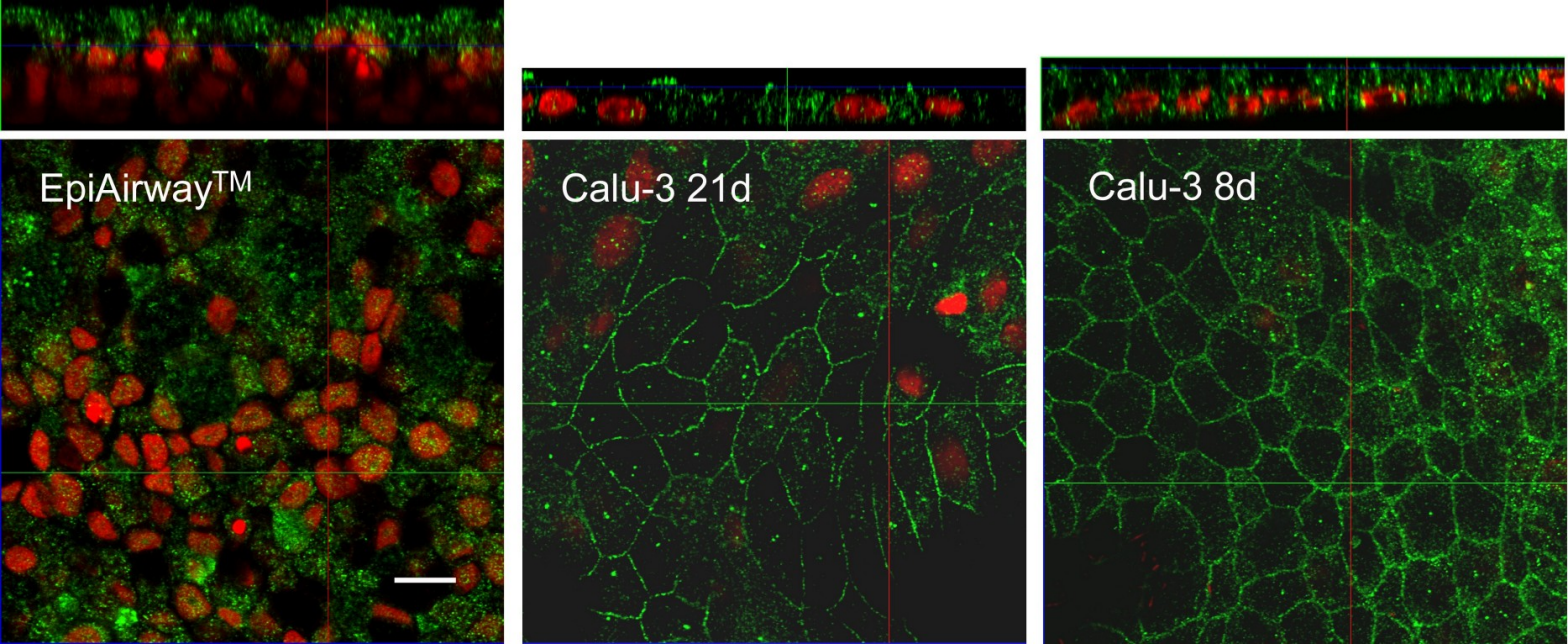
Immunolocalization of OCTN2 transporter in EpiAirway^™^ and Calu-3. Confocal laser scanning microscopy of monolayers immunolabelled for OCTN2 transporter (green) is shown. Nuclei were stained with propidium iodide (red). Single XY scans of EpiAirway^™^ and Calu-3 cultured under ALI conditions for 8d or 21d, as indicated, are shown; XZ sections of the planes are shown on the top. Representative images from three independent experiments are shown. Bar, 10 μm.

As for OCTN2, despite the positivity of the staining in EpiAirway^™^, the signal was not definitely localized; the same analysis in Calu-3 at both 8d and 21d ALI revealed, instead, a continuous staining around cell borders on the lateral plasma membrane (Fig 6), supporting the basolateral localization of the transporter, pointed out by functional data.

**Fig 7:**
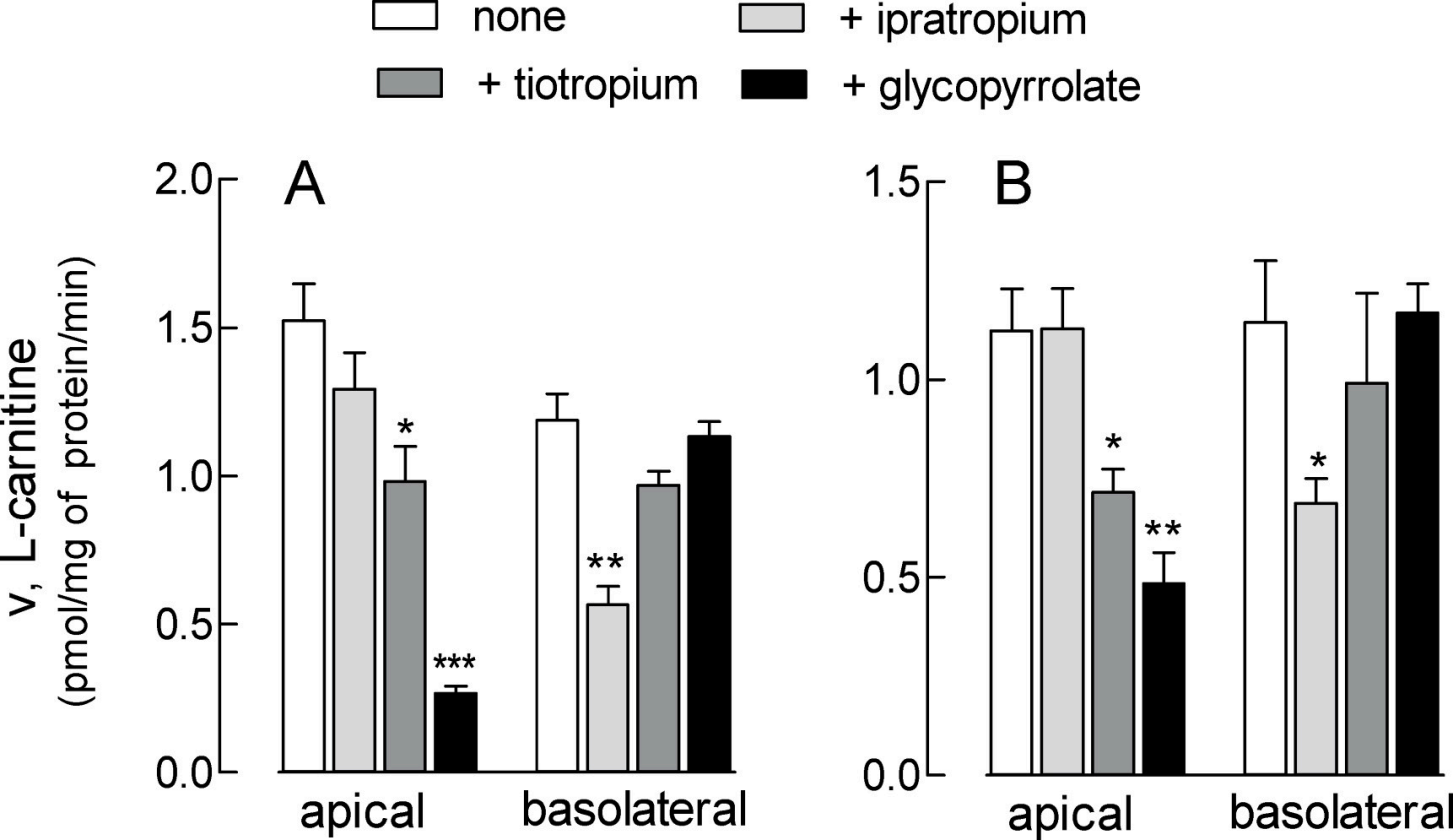
Effect of ipratropium, tiotropium and glycopyrrolate on L-carnitine uptake in EpiAirway^™^ and Calu-3 cells. Monolayers of EpiAirway^™^ (A) and Calu-3 cells grown under air-liquid interface (ALI) conditions for 8d (B) were washed with EBSS and incubated for 30 min, either at the apical or the basolateral compartment, in EBSS containing [^3^H]carnitine (1 μM; 2 μCi/ml) in the absence (none) or in the presence of 200 μM ipratropium, tiotropium, or glycopyrrolate, as indicated. Bars represent the mean ± SEM of three (EpiAirway^™^) or four (Calu-3 cells) independent determinations. *p<0.05, **p<0.01, ***p<0.001 vs none. Original data are available at osf.io (DOI 10.17605/OSF.IO/Z3UB5).

In order to examine the role of L-carnitine transporters in drug delivery in the airways, the effect of three bronchodilators (ipratropium, tiotropium, and glycopyrrolate) was investigated both in EpiAirway^™^ and in Calu-3 cells at 8d. As shown in Fig 7, glycopyrrolate markedly reduced apical L-carnitine uptake in both EpiAirway^™^ (panel A) and Calu-3 (panel B); also tiotropium was effective at the apical side of both models, although to a lesser extent, while ipratropium had no effect. This latter, however, was the sole able to inhibit L-carnitine uptake at the basolateral side, where the other two drugs were, instead, completely ineffective. These results indicate that tiotropium and glycopyrrolate interact with ATB^0,+^ at the apical side of airway epithelia, while ipratropium is substrate of OCTN2.

**Fig 8:**
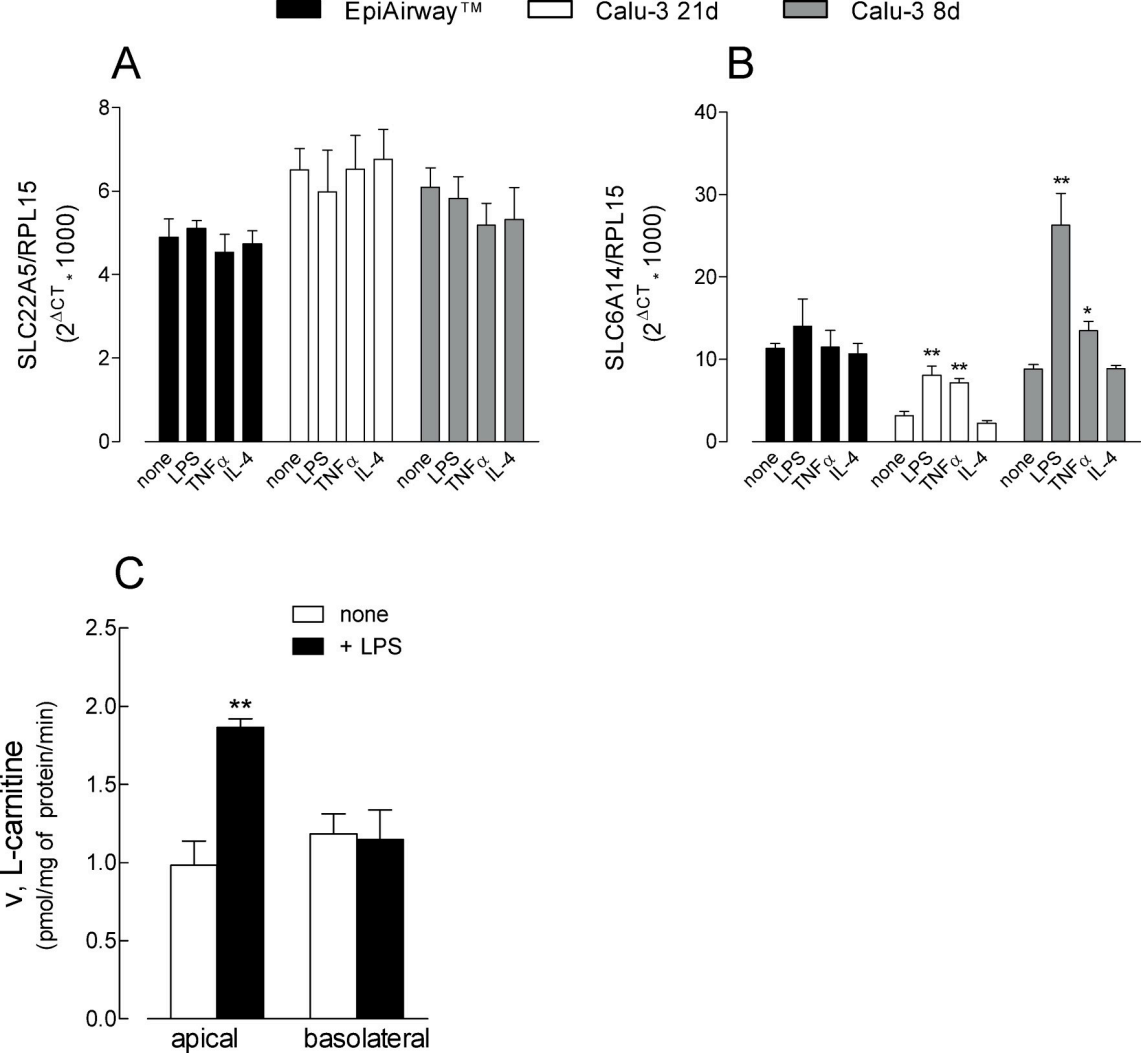
Effect of inflammatory stimuli on the expression of OCTN2 and ATB^0,+^ in EpiAirway^™^ and Calu-3 cells cultured under ALI conditions for 8d or 21d. LPS (10 μg/ml), TNFα (10 ng/ml) or IL-4 (10 ng/ml) were added to the culture medium for 24 h. The expression of SLC22A5/OCTN2 (A) and SLC6A14/ATB^0,+^ (B) was determined by means of RT-qPCR analysis, as described in Fig 3. Data are means ± SEM of four independent experiments. L-Carnitine uptake was measured in Calu-3 at 8d (C), as described in Fig 1; data are means ± SEM of three independent experiments. *p<0.05, **p<0.01 vs none. Original data are available at osf.io (DOI 10.17605/OSF.IO/Z3UB5).

Given the involvement of these transporter in drug delivery, we lastly evaluated the impact of inflammatory stimuli on their expression and function. To this end, the expression of ATB^0,+^ and OCTN2 in EpiAirway^™^ and in Calu-3 cells has been investigated under inflammatory conditions, i.e in the presence of microbe-specific stimulus lipopolysaccharides (LPS) or Tumor necrosis factor α (TNFα); the effect of the anti-inflammatory Interleukin 4 (IL-4) was also tested. Results presented in Fig 8 show that the expression of SLC22A5/OCTN2 was unaffected by any of the experimental condition adopted in either cell model (panel A). On the contrary, the exposure of cells to either LPS or TNFα markedly enhanced the expression of SLC6A14/ATB^0,+^ in Calu-3 cells cultured for both 21d and 8d; a slight, although not significant increase was also observed in EpiAirway^™^ in the presence of LPS (panel B) In all cell models, IL-4 was ineffective, suggesting that the transporter is a target only of proinflammatory stimuli. Consistently, L-carnitine uptake (panel C) was significantly upregulated by LPS at the apical, but not at the basolateral side of Calu-3 cells at 8d, when ATB^0,+^ reached the maximal expression.

## Discussion

This study is the first to evaluate the expression and activity of L-carnitine transporters in EpiAirway^™^, an organotypic model of human airway epithelium. To date, most of the studies dealing with the expression of membrane transporters in human respiratory epithelium have been performed in cell lines, such as Calu-3, BEAS-2B and 16HBE14- cells; Calu-3 cells, in particular, are considered a reference model in studies of pulmonary drug absorption [31]. These models, however, are immortalized or transformed cells, that are suspected to display biological functions deviating from those of primary respiratory cells obtained *ex vivo* from tissues [32]. Recently, ready-to-use, three-dimensional cultures of primary airway cells have been developed, such as EpiAirway^™^, that represent a promising *in vitro* model for human lung. By comparing L-carnitine transport in EpiAirway^™^ and Calu-3 cells, we demonstrate here that it is mediated in both models by ATB^0,+^ at the apical side and by OCTN2 on the basolateral membrane.

Really, an immunohistochemistry analysis recently performed in lung tissues from healthy and COPD patients detects OCTN2 on the apical and lateral side of the epithelial cells in the bronchial region and in cells lining the bronchioles [33]; similarly, the transporter has been shown on the apical surface of ALI Calu-3 layers [15]. In our hands, conversely, the immunocytochemical images indicate a prevalent expression of the transporter on the lateral plasma membrane, data completely consistent with results of transport experiments. Although we do not feel able to exclude an also apical expression of OCTN2 on the basis of immunocytochemistry, the functional analysis seems to limit the activity of the transporter to the basolateral side of both cell models.

Similarly, transport data and immunocytochemical staining converge in proving the apical localization of ATB^0,+^. This transporter appears, indeed, highly expressed and operative exclusively at the apical side of both EpiAirway^™^ and Calu-3 layers maintained at ALI for 8 d. Interestingly, the uptake of carnitine through ATB^0,+^, as well as the expression of the transporter, are no longer appreciable in fully differentiated 21d monolayers, confirming the finding that the biological features of Calu-3 cells may be modified by the culture conditions (e.g. air or liquid interface and time in culture) [34]; in this context, we suggest to include ATB^0,+^ expression in the list of parameters that vary along with the duration of the culture.

Beyond transporting L-carnitine, ATB^0,+^ and OCTN2 have significant potential as delivery systems for amino acid-based drugs and prodrugs [21,35]. OCTN2 in particular is described to interact with inhaled drugs e.g. muscarinic antagonists and β-adrenergic agonists cationic bronchodilators [36]; moreover, this transporter offers an efficient mean to deliver drugs or drug-loaded nanoparticles conjugated to carnitine [37]. Results about this issue are, however, controversial. A previous study performed in OCTN2-transfected HEK293 cells and in OCTN2-silenced BEAS-2B reports that tiotropium and ipratropium, anti-cholinergic drugs approved for treatment of asthma and COPD [38], are taken up primarily by OCTN2 [18]. On the other hand, a recent permeability study in Calu-3 layers shows that L-carnitine does not alter ipratropium transepithelial transport, ruling out an involvement of OCTN2 in the drug trafficking across the monolayer [39]. In this dispute, our results in EpiAirway^™^ and Calu-3 cells point to the interaction of ipratropium, but not of tiotropium, with OCTN2; in our hands, tiotropium rather behaves as inhibitor of ATB^0,+^ like the other anti-cholinergic bronchodilator glycopyrrolate, which is the most effective on this transporter. The capacity of ATB^0,+^ to interact with drugs gains particular relevance when considering the ability of inflammatory stimuli to modulate the expression of this transporter. Indeed, it has been recently suggested that ATB^0,+^ typically localizes in places where the body interfaces with microbes, such as lung and colon, where it is probably involved in reducing available nutrients to bacteria; consistently, the transporter is up-regulated in inflammatory states, such as ulcerative colitis, Crohn’s disease and colon cancer [20]. Moreover, genome-wide association studies has recently identified SLC6A14/ATB^0,+^ as a genetic modifier of lung disease severity in cystic fibrosis, providing a mechanism by which it regulates Pseudomonas aeruginosa attachment to human bronchial epithelial cells [40]. In that study, Di Paola *et al*. demonstrated an enhancement of SLC6A14 expression by LPS in Calu-3 cells cultured on plasticware. In line with those observations, we here show a marked induction of SLC6A14/ATB^0,+^ upon exposure of polarized Calu-3 cells to inflammatory stimuli, like TNFα and LPS; this latter, in particular, induced an impressive expression of the transporter in Calu-3 ALI consistent with the stimulation of L-carnitine transport at the apical side. Since IL-4 had no effect on SLC6A14 expression, we can conclude that this transporter is a target of Th1 cytokines. This response was not evidenced in EpiAirway^™^, where the slight induction of SLC6A14 expression did not reach statistical significance; whether this discrepancy reflects the different structure of the layer, pseudostratified in EpiAirway^™^ and monolayer in Calu-3, or rather to the different cellular composition of the two tissues, remains to be determined. When addressing the effects of the same stimuli on OCTN2, we did not highlight any modulation of SLC22A5 gene expression nor of L-carnitine transport at the basolateral side of either EpiAirway^™^ or Calu-3. These results contradict the findings by Mukherjee *et al*. [41], showing that the exposure of polarized Calu-3 cells to LPS as well as to aeroallergen house dust mite (HDM) induces an impressive stimulation of OCTN2/SLC22A5. To this concern, however, a recent contribution by Li *et al*. [42] demonstrates that the treatment with LPS even down-regulates OCTN1/2 expression and activity in alveolar A549 cells. Further studies will be required to define the effects of proinflammatory stimuli on OCTN2, as well as to investigate the role of L-carnitine transporters under inflammatory conditions.

## Conclusions

In conclusion, our findings indicate that ATB^0,+^ transporter localizes at the apical side of EpiAirway^™^, while OCTN2 at the basolateral. A similar pattern is displayed in Calu-3 monolayers cultured at ALI condition only for short time, since the activity of ATB^0,+^ is no more detectable when the culture is prolonged to 21 d. In both models, the two transporters differentially interact with bronchodilators, in particular glycopyrrolate and tiotropium with ATB^0,+^ and ipratropium with OCTN2. These findings can open new fields of investigation on the mechanisms regulating the disposition of these drugs through the epithelial layer, and, in this context, EpiAirway^™^ provides a reliable tool for studies in the field of pulmonary drug delivery.

## Supporting information

S1 Fig Trans-epitelial electrical resistance (TEER) and mannitol permeability (*P_app_*) of EpiAirway^™^ and Calu-3 cells. TEER and *P*_*app*_ values were measured in EpiAirway^™^ and Calu-3 cultured under ALI conditions for 8d or 21d (see Methods). Individual data points are shown, with indicated the mean ± SEM of replicates. Original data are available at osf.io (DOI 10.17605/OSF.IO/Z3UB5).
(TIF)

## Abbreviations

ALI: air-liquid interface
ATB^0,+^: amino acid transporter B^0,+^
3D: three-dimensional
EBSS: Earle’s Balanced Salt Solution
OCTN: novel organic cation transporter
PBS: phosphate-buffered saline solution
TEER: transepithelial electrical resistance

## References

[1] Longo N, Frigeni M, Pasquali M (2016) Carnitine transport and fatty acid oxidation. Biochim Biophys Acta 1863: 2422–2435. 10.1016/j.bbamcr.2016.01.023 26828774 PMC4967041

[2] Ferreira GC, McKenna MC (2017) L-Carnitine and Acetyl-L-carnitine Roles and Neuroprotection in Developing Brain. Neurochem Res 42: 1661–1675. 10.1007/s11064-017-2288-7 28508995 PMC5621476

[3] Gulcin I (2006) Antioxidant and antiradical activities of L-carnitine. Life Sci 78: 803–811. 10.1016/j.lfs.2005.05.103 16253281

[4] Ribas GS, Manfredini V, de Marco MG, Vieira RB, Wayhs CY, Vanzin CS, et al (2010) Prevention by L-carnitine of DNA damage induced by propionic and L-methylmalonic acids in human peripheral leukocytes in vitro. Mutat Res 702: 123–128. 10.1016/j.mrgentox.2010.07.008 20659584

[5] Tamai I, Ohashi R, Nezu J, Yabuuchi H, Oku A, Shimane M, et al (1998) Molecular and functional identification of sodium ion-dependent, high affinity human carnitine transporter OCTN2. J Biol Chem 273: 20378–20382. 10.1074/jbc.273.32.20378 9685390

[6] Wu X, Huang W, Prasad PD, Seth P, Rajan DP, Leibach FH, et al (1999) Functional characteristics and tissue distribution pattern of organic cation transporter 2 (OCTN2), an organic cation/carnitine transporter. J Pharmacol Exp Ther 290: 1482–1492. 10454528

[7] Salomon JJ, Gausterer JC, Selo MA, Hosoya KI, Huwer H, Schneider-Daum N, et al (2019) OCTN2-Mediated Acetyl-l-Carnitine Transport in Human Pulmonary Epithelial Cells In Vitro. Pharmaceutics 11. 10.3390/pharmaceutics11080396 PMC6723908 31394757

[8] Wagner CA, Lukewille U, Kaltenbach S, Moschen I, Broer A, Risler T, et al (2000) Functional and pharmacological characterization of human Na(+)-carnitine cotransporter hOCTN2. Am J Physiol Renal Physiol 279: F584–591. 10.1152/ajprenal.2000.279.3.F584 10966938

[9] Ingoglia F, Visigalli R, Rotoli BM, Barilli A, Riccardi B, Puccini P, et al (2017) Human macrophage differentiation induces OCTN2-mediated L-carnitine transport through stimulation of mTOR-STAT3 axis. J Leukoc Biol 101: 665–674. 10.1189/jlb.1A0616-254R 27733576

[10] Pochini L, Galluccio M, Scalise M, Console L, Indiveri C (2019) OCTN: A Small Transporter Subfamily with Great Relevance to Human Pathophysiology, Drug Discovery, and Diagnostics. SLAS Discov 24: 89–110. 10.1177/2472555218812821 30523710

[11] Endter S, Francombe D, Ehrhardt C, Gumbleton M (2009) RT-PCR analysis of ABC, SLC and SLCO drug transporters in human lung epithelial cell models. J Pharm Pharmacol 61: 583–591. 10.1211/jpp/61.05.0006 19405996

[12] Bundey RA, Insel PA (2003) Quantification of adenylyl cyclase messenger RNA by real-time polymerase chain reaction. Anal Biochem 319: 318–322. 10.1016/s0003-2697(03)00325-7 12871728

[13] Sakamoto A, Matsumaru T, Yamamura N, Suzuki S, Uchida Y, Tachikawa M, et al (2015) Drug Transporter Protein Quantification of Immortalized Human Lung Cell Lines Derived from Tracheobronchial Epithelial Cells (Calu-3 and BEAS2-B), Bronchiolar-Alveolar Cells (NCI-H292 and NCI-H441), and Alveolar Type II-like Cells (A549) by Liquid Chromatography-Tandem Mass Spectrometry. J Pharm Sci 104: 3029–3038. 10.1002/jps.24381 25690838

[14] Salomon JJ, Endter S, Tachon G, Falson F, Buckley ST, Ehrhardt C (2012) Transport of the fluorescent organic cation 4-(4-(dimethylamino)styryl)-N-methylpyridinium iodide (ASP+) in human respiratory epithelial cells. Eur J Pharm Biopharm 81: 351–359. 10.1016/j.ejpb.2012.03.001 22426135

[15] Mukherjee M, Pritchard DI, Bosquillon C (2012) Evaluation of air-interfaced Calu-3 cell layers for investigation of inhaled drug interactions with organic cation transporters in vitro. Int J Pharm 426: 7–14. 10.1016/j.ijpharm.2011.12.036 22265910

[16] Giacomini KM, Huang SM, Tweedie DJ, Benet LZ, Brouwer KL, Chu X, et al (2010) Membrane transporters in drug development. Nat Rev Drug Discov 9: 215–236. 10.1038/nrd3028 20190787 PMC3326076

[17] Nakanishi T, Hasegawa Y, Haruta T, Wakayama T, Tamai I (2013) In vivo evidence of organic cation transporter-mediated tracheal accumulation of the anticholinergic agent ipratropium in mice. J Pharm Sci 102: 3373–3381. 10.1002/jps.23603 23686692

[18] Nakamura T, Nakanishi T, Haruta T, Shirasaka Y, Keogh JP, Tamai I (2010) Transport of ipratropium, an anti-chronic obstructive pulmonary disease drug, is mediated by organic cation/carnitine transporters in human bronchial epithelial cells: implications for carrier-mediated pulmonary absorption. Mol Pharm 7: 187–195. 10.1021/mp900206j 20020740

[19] Nakanishi T, Hatanaka T, Huang W, Prasad PD, Leibach FH, Ganapathy ME, et al (2001) Na+- and Cl—coupled active transport of carnitine by the amino acid transporter ATB(0,+) from mouse colon expressed in HRPE cells and Xenopus oocytes. J Physiol 532: 297–304. 10.1111/j.1469-7793.2001.0297f.x 11306651 PMC2278546

[20] Broer S, Fairweather SJ (2018) Amino Acid Transport Across the Mammalian Intestine. Compr Physiol 9: 343–373. 10.1002/cphy.c170041 30549024

[21] Hatanaka T, Haramura M, Fei YJ, Miyauchi S, Bridges CC, Ganapathy PS, et al (2004) Transport of amino acid-based prodrugs by the Na+- and Cl(-) -coupled amino acid transporter ATB0,+ and expression of the transporter in tissues amenable for drug delivery. J Pharmacol Exp Ther 308: 1138–1147. 10.1124/jpet.103.057109 14617696

[22] Galietta LJ, Musante L, Romio L, Caruso U, Fantasia A, Gazzolo A, et al (1998) An electrogenic amino acid transporter in the apical membrane of cultured human bronchial epithelial cells. Am J Physiol 275: L917–923. 10.1152/ajplung.1998.275.5.L917 9815109

[23] Rudnick G, Kramer R, Blakely RD, Murphy DL, Verrey F (2014) The SLC6 transporters: perspectives on structure, functions, regulation, and models for transporter dysfunction. Pflugers Arch 466: 25–42. 10.1007/s00424-013-1410-1 24337881 PMC3930102

[24] Haghi M, Ong HX, Traini D, Young P (2014) Across the pulmonary epithelial barrier: Integration of physicochemical properties and human cell models to study pulmonary drug formulations. Pharmacol Ther 144: 235–252. 10.1016/j.pharmthera.2014.05.003 24836727

[25] Berube K, Prytherch Z, Job C, Hughes T (2010) Human primary bronchial lung cell constructs: the new respiratory models. Toxicology 278: 311–318. 10.1016/j.tox.2010.04.004 20403407

[26] Chemuturi NV, Hayden P, Klausner M, Donovan MD (2005) Comparison of human tracheal/bronchial epithelial cell culture and bovine nasal respiratory explants for nasal drug transport studies. J Pharm Sci 94: 1976–1985. 10.1002/jps.20404 16052562

[27] Koepsell H, Lips K, Volk C (2007) Polyspecific organic cation transporters: structure, function, physiological roles, and biopharmaceutical implications. Pharm Res 24: 1227–1251. 10.1007/s11095-007-9254-z 17473959

[28] Closs EI, Simon A, Vekony N, Rotmann A (2004) Plasma membrane transporters for arginine. J Nutr 134: 2752S–2759S; discussion 2765S-2767S. 10.1093/jn/134.10.2752S 15465780

[29] Rotoli BM, Barilli A, Visigalli R, Ferrari F, Dall'Asta V (2020) y+LAT1 and y+LAT2 contribution to arginine uptake in different human cell models: Implications in the pathophysiology of Lysinuric Protein Intolerance. J Cell Mol Med 24: 921–929. 10.1111/jcmm.14801 31705628 PMC6933409

[30] Gazzola GC, Dall'Asta V, Franchi-Gazzola R, White MF (1981) The cluster-tray method for rapid measurement of solute fluxes in adherent cultured cells. Anal Biochem 115: 368–374. 10.1016/0003-2697(81)90019-1 7304965

[31] Mathia NR, Timoszyk J, Stetsko PI, Megill JR, Smith RL, Wall DA (2002) Permeability characteristics of calu-3 human bronchial epithelial cells: in vitro-in vivo correlation to predict lung absorption in rats. J Drug Target 10: 31–40. 10.1080/10611860290007504 11996084

[32] Akhtar US, Scott JA, Chu A, GJ E (2010) In vivo and In vitro Assessment of Particulate Matter Toxicology In: Zereini F. WC, editor. Urban Airborne Particulate Matter Environmental Science and Engineering (Environmental Engineering): Springer, Berlin, Heidelberg pp. 427–449.

[33] Berg T, Hegelund-Myrback T, Ockinger J, Zhou XH, Brannstrom M, Hagemann-Jensen M, et al (2018) Expression of MATE1, P-gp, OCTN1 and OCTN2, in epithelial and immune cells in the lung of COPD and healthy individuals. Respir Res 19: 68 10.1186/s12931-018-0760-9 29678179 PMC5910606

[34] Kreft ME, Jerman UD, Lasic E, Hevir-Kene N, Rizner TL, Peternel L, et al (2015) The characterization of the human cell line Calu-3 under different culture conditions and its use as an optimized in vitro model to investigate bronchial epithelial function. Eur J Pharm Sci 69: 1–9. 10.1016/j.ejps.2014.12.017 25555374

[35] Koepsell H, Endou H (2004) The SLC22 drug transporter family. Pflugers Arch 447: 666–676. 10.1007/s00424-003-1089-9 12883891

[36] Bosquillon C (2010) Drug transporters in the lung—do they play a role in the biopharmaceutics of inhaled drugs? J Pharm Sci 99: 2240–2255. 10.1002/jps.21995 19950388

[37] Kou L, Sun R, Ganapathy V, Yao Q, Chen R (2018) Recent advances in drug delivery via the organic cation/carnitine transporter 2 (OCTN2/SLC22A5). Expert Opin Ther Targets 22: 715–726. 10.1080/14728222.2018.1502273 30016594

[38] Gosens R, Gross N (2018) The mode of action of anticholinergics in asthma. Eur Respir J 52. 10.1183/13993003.01247-2017 PMC6340638 30115613

[39] Panduga V, Stocks MJ, Bosquillon C (2017) Ipratropium is 'luminally recycled' by an inter-play between apical uptake and efflux transporters in Calu-3 bronchial epithelial cell layers. Int J Pharm 532: 328–336. 10.1016/j.ijpharm.2017.08.112 28855136

[40] Di Paola M, Park AJ, Ahmadi S, Roach EJ, Wu YS, Struder-Kypke M, et al (2017) SLC6A14 Is a Genetic Modifier of Cystic Fibrosis That Regulates Pseudomonas aeruginosa Attachment to Human Bronchial Epithelial Cells. MBio 8. 10.1128/mBio.02073-17 PMC5736915 29259090

[41] Mukherjee M, Cingolani E, Pritchard DI, Bosquillon C (2017) Enhanced expression of Organic Cation Transporters in bronchial epithelial cell layers following insults associated with asthma—Impact on salbutamol transport. Eur J Pharm Sci 106: 62–70. 10.1016/j.ejps.2017.05.052 28549677

[42] Li D, Qi C, Zhou J, Wen Z, Zhu X, Xia H, et al (2019) LPS-induced inflammation delays the transportation of ASP(+) due to down-regulation of OCTN1/2 in alveolar epithelial cells. J Drug Target: 1–11. 10.1080/1061186X.2019.1678169 31591905

