# Correction: Functional analysis of OCTN2 and ATB^0,+^ in normal human airway epithelial cells

**DOI:** 10.1371/journal.pone.0230456

**Published:** 2020-03-10

**Authors:** Bianca Maria Rotoli, Rossana Visigalli, Amelia Barilli, Francesca Ferrari, Massimiliano G. Bianchi, Maria Di Lascia, Benedetta Riccardi, Paola Puccini, Valeria Dall’Asta

There is an error in reference 12. The correct reference is: Ingoglia F, Visigalli R, Rotoli BM, Barilli A, Riccardi B, Puccini P, et al. (2016) Functional activity of L-carnitine transporters in human airway epithelial cells. Biochim Biophys Acta. 1858(2):210–9. 10.1016/j.bbamem.2015.11.013.
