# Supplementary figures and images for: Functional analysis of OCTN2 and ATB0,+ in normal human airway epithelial cells
Source: PLoS One. 2020 Feb 6;15(2):e0228568. doi: 10.1371/journal.pone.0228568 (PMC7004352; doi:10.1371/journal.pone.0228568)

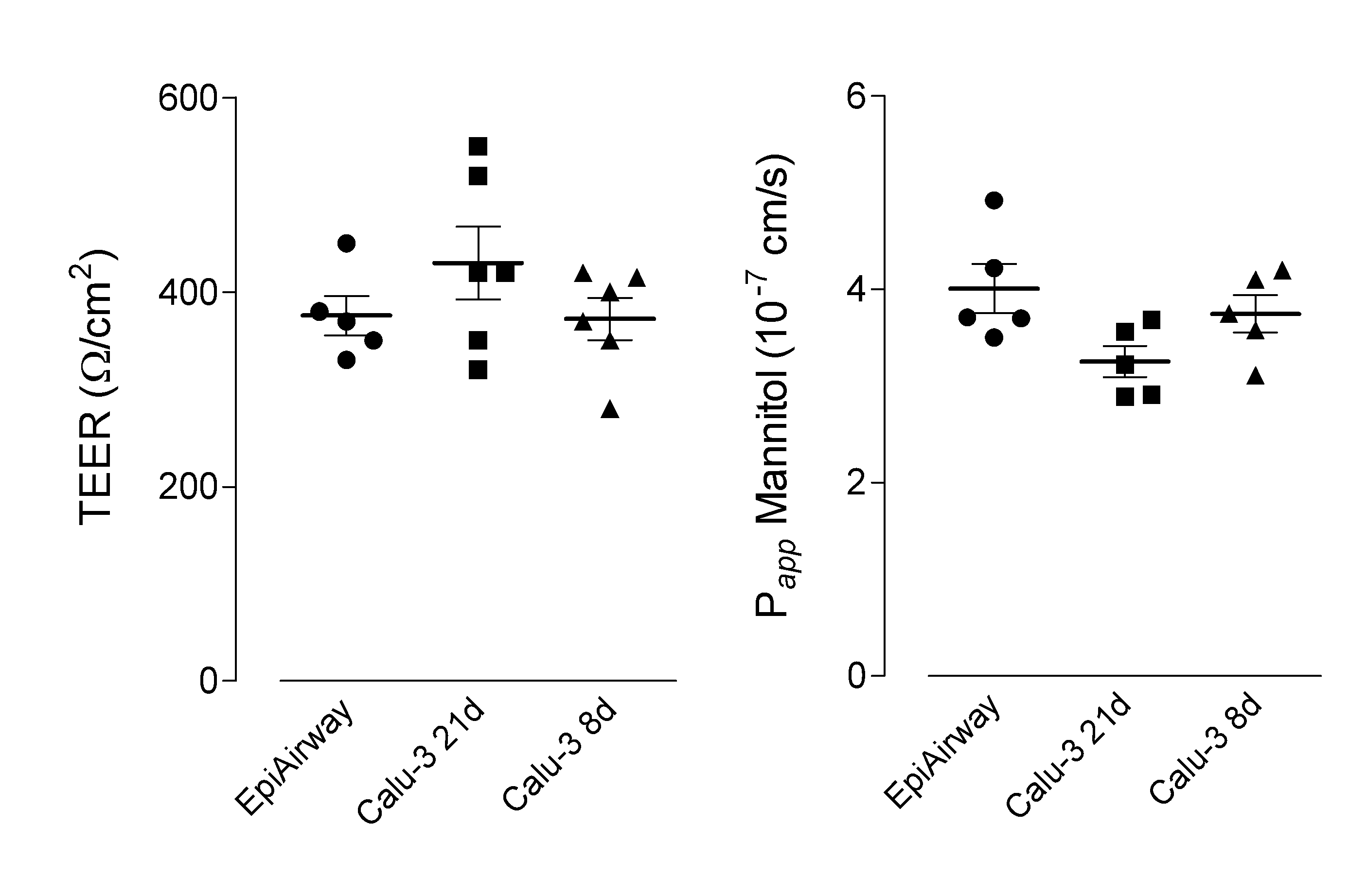

Supplement: S1 Fig — TEER and Papp values were measured in EpiAirway™ and Calu-3 cultured under ALI conditions for 8d or 21d (see Methods). Individual data points are shown, with indicated the mean ± SEM of replicates. Original data are available at osf.io (DOI 10.17605/OSF.IO/Z3UB5). (TIF) [file pone.0228568.s001.tif]
